# Advancing towards a Practical Magnesium Ion Battery

**DOI:** 10.3390/ma14237488

**Published:** 2021-12-06

**Authors:** Alejandro Medina, Carlos Pérez-Vicente, Ricardo Alcántara

**Affiliations:** Department of Inorganic Chemistry, Instituto Universitario de Investigación en Química Fina y Nanoquímica (IUNAN), Faculty of Sciences, Campus de Rabanales, University of Córdoba, Edificio Marie Curie, 14071 Córdoba, Spain; q42mejaa@uco.es (A.M.); iq3pevic@uco.es (C.P.-V.)

**Keywords:** post-lithium batteries, magnesium-ion battery, intercalation, solid electrolyte

## Abstract

A post-lithium battery era is envisaged, and it is urgent to find new and sustainable systems for energy storage. Multivalent metals, such as magnesium, are very promising to replace lithium, but the low mobility of magnesium ion and the lack of suitable electrolytes are serious concerns. This review mainly discusses the advantages and shortcomings of the new rechargeable magnesium batteries, the future directions and the possibility of using solid electrolytes. Special emphasis is put on the diversity of structures, and on the theoretical calculations about voltage and structures. A critical issue is to select the combination of the positive and negative electrode materials to achieve an optimum battery voltage. The theoretical calculations of the structure, intercalation voltage and diffusion path can be very useful for evaluating the materials and for comparison with the experimental results of the magnesium batteries which are not hassle-free.

## 1. Introduction

**Figure 1:**
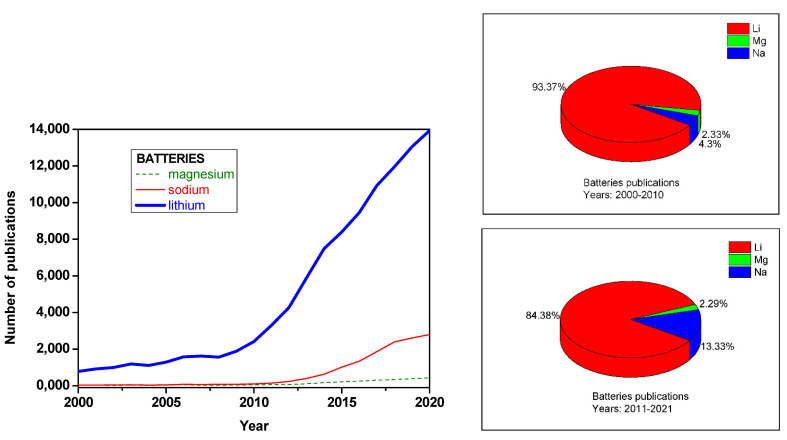
Comparison of the number of publications for LIB and post-lithium batteries (sodium and magnesium). **Left**: number of publications per year about magnesium, sodium and lithium batteries from 2000 to 2020. **Right**: relative amounts of publications in the periods 2000–2010 and 2011–2021 (up to October). The data were obtained from the Web of Science.

Rechargeable batteries are essential components of many electrical devices and have a great development prospect, particularly in vehicles and renewable energies. Lithium batteries dominate a large market niche, but these batteries have limits, such as safety concerns and high economic cost [1]. Magnesium could be at the front of the race for seeking new batteries beyond lithium-ion technology. Mainly due to large natural abundance, low price and divalent character, magnesium could replace lithium in the batteries. The batteries based on the reversible intercalation of magnesium ion into host materials are usually referred as “magnesium ion battery” (MIB), analogously to Li-ion battery (LIB). Compared to lithium and sodium batteries, the development of rechargeable magnesium batteries is more difficult, and even the investigation is plenty of difficulties and the number of publications is relatively reduced (Figure 1). The tendency is to increase the relative proportion of efforts devoted to investigating post-lithium batteries. Probably, the “publications peak” for lithium and sodium will be in a near future, while magnesium is still in its infancy.

Unlikely to commercial LIB, in many papers about MIB the intercalation of magnesium-ion happens only at the positive electrode, and the negative electrode is not a Mg-intercalation compound but Mg metal. In fact, the use of Mg metal in rechargeable batteries could be very advantageous compared to Li metal, particularly in terms of volumetric capacity (3837 mAh cm^−3^) and safety. Mg metal is much less reactive than Li and Na in air atmosphere, and Mg electrode is less prone to dendrite formation [2]. However, several challenges, still, must yet be addressed before commercialization of MIB. For example, there is not an adequate electrolyte solution compatible with both electrodes. For example, in many scientific papers activated carbon is used as negative electrode (instead of Mg) to study the Mg-intercalation just in the positive electrode, although this approach is valid for research purposes only.

**Figure 2:**
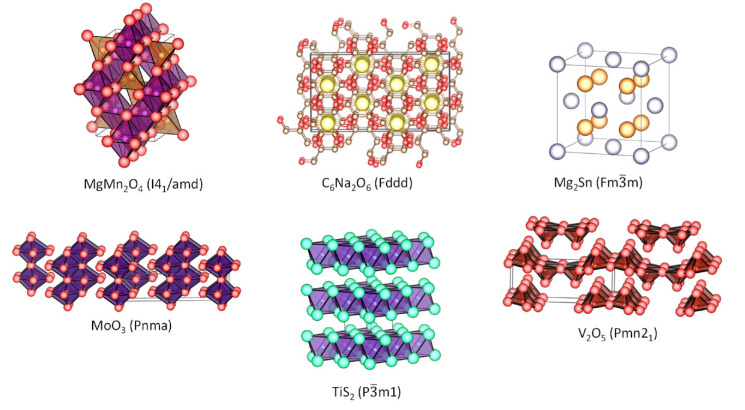
Structures of selected Mg-intercalation compounds with several dimensionalities.

To achieve high voltage and high energy density in the battery, the (low-voltage) negative electrode of Mg should be combined with a high-voltage positive electrode, and for that purpose several candidates have been explored in the literature (Figure 2). Unfortunately, many compounds and structures which are very suitable for reversible intercalation of lithium and/or sodium have poor capacity in magnesium cell, and the main reasons for that could be the strong electrostatic repulsions between cations and the anion-cation attractions. It is generally believed that the electrostatic interactions hinder the diffusion of magnesium ion in the solids. In addition, the diffusion of magnesium in many solids remains unexplored.

Ion intercalation is associated with changes in the oxidation state of the atoms in the intercalation compound. Not only the electrostatic repulsions and attractions influence on the intercalation and mobility of magnesium, but also the redistribution of the charges. According to the second Pauling’s rule, or the electrostatic rule, the charge of the anion must be balanced by an equal opposite charge centered on the surrounding cations [3]. To satisfy this Pauling’s rule for partially covalent/ionic crystal, and with no dramatic change of the bonding and structure after intercalation of magnesium, the adjacent transition metal ion must change its oxidation state by two electrons [4]. The multielectron redox processes could drive to ionic radii change and structure change. These facts limit the compounds which are suitable as hosts for intercalation of magnesium and others multivalent cations. Nevertheless, there is a chance to find materials with rapid Mg-mobility and reasonable migration barriers at room temperature [5]. Thus, in principle, vanadium, molybdenum and manganese are between the most promising multivalent elements which should be found in Mg-intercalation compounds, but nevertheless other elements could be used as dopants (e.g., aluminum and iron).

Many efforts are being devoted to developing suitable electrolytes. The electrolyte must possess high ionic conductivity and favor the migration of magnesium ion and the electron transfer at the electrolyte/electrode interface. Liquid electrolytes very often lead to high interfacial impedance, overpotential for Mg plating/stripping, poor coulombic efficiency and safety problems. As due to the strong electrostatic interactions, desolvation of Mg^2+^ can lead to overpotential and failure to intercalate in the electrode. One of the main drawbacks is that many common electrolytes form a passivating layer on the surface of Mg metal that blocks the diffusion of magnesium ion and hinders the reversible plating. In contrast to the solid electrolyte interphase (SEI) formed on Li metal which is Li^+^-conducting, the film formed on Mg is not Mg^2+^-conducting for many liquid electrolytes, and the resulting charge transfer resistance is very high. Many conventional solvents used in LIB cannot be efficiently employed in MIB [6], such as carbonates [7]. Magnesium forms strong bonds with high dissociation energy and, consequently, the dissociation of the magnesium-solvent complex is very sluggish for many electrolyte solutions. Besides that, some electrolytes that exhibit good properties for reversible plating/stripping of Mg are decomposed at the operating voltage of many positive electrodes, or even they corrode the stainless steel used as current collector [8]. For example, a certain amount of MgCl_2_ in the electrolyte solution can be beneficial for the reversible platting of Mg, but it could be harmful for other components of the battery. The breakage of the strong Mg-Cl bond before Mg-intercalation into the host solid also can be a drawback. In addition, the role played by the traces of water and other impurities in the electrolyte solution is not yet clear. Trying to avoid the problems inherent to the volatility of many organic solvents, systems using ionic liquids have been reported, but the reproducibility and reversibility of Mg plating/tripping in these systems is not yet conformed [9,10], and the compatibility of the ionic liquid with the high-voltage electrodes could be doubtful.

**Figure 3:**
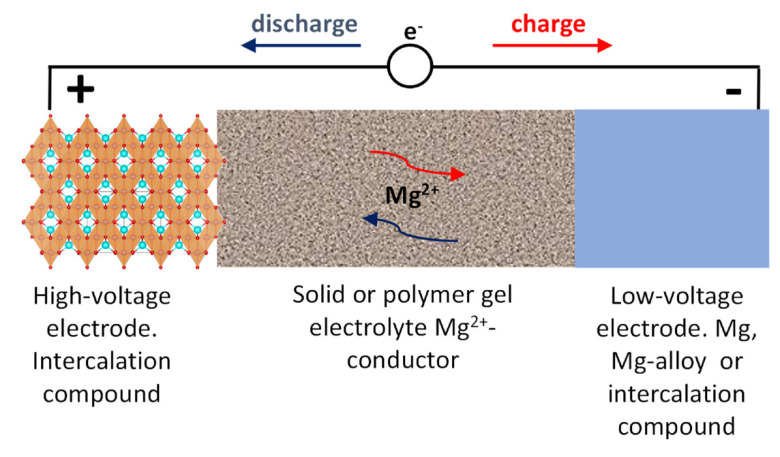
Schematic representation (not to scale) of an all-solid state magnesium battery.

Using a solid state electrolyte could be an alternative way to develop the future rechargeable MIB [11,12]. The main concept behind an all-solid-state MIB is drawn in Figure 3. The positive electrode, or cathode, should (de)intercalate magnesium at high voltage. The negative electrode, or anode, must operate at low voltage. The solid electrolyte must be a good Mg-ion conductor and non-conductor of electrons, stable at the operation voltages of the two electrode materials and fabricated with a minimum thickness. The weighs of all these components should be balanced, and the electrodes should be well connected with the electrolyte to minimize the interface resistance. The volume changes of the battery components during charge-discharge could affect to the contacts and interfaces and, thus, the volume changes of the electrodes and the electrolyte should be minimum. These batteries based on solid electrolyte could be safer and easier of fabrication. Unfortunately, the slow diffusion rate of magnesium ion in the solid state is another challenge [4]. Although not many papers have been reported about Mg^2+^-solid electrolytes, we think that a huge interest could emerge in the forthcoming years.

Compared to other review papers on MIB [13,14,15,16,17,18,19,20], this article aims to include both the latest updates on electrode materials and to focus on the critical assessment of the battery components which could provide and advancement towards an all-solid-state magnesium battery. Besides that, the theoretical calculations and the experimental results are critically confronted, and previously unpublished theoretical calculations are reported here.

**Figure 4:**
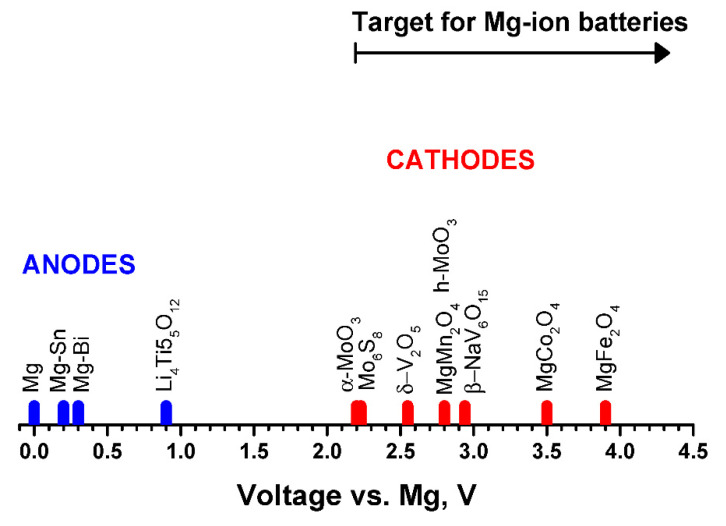
Working voltages for several electrode materials of MIB. The theoretically calculated values are employed here when these data are available in the literature or, alternatively, the experimental values are used.

The working voltages reported for significant electrode materials are shown in Figure 4. In a MIB containing Mg-metal or Mg-alloy as negative electrode, the initial composition of the positive electrode could be Mg-free, and then the electrochemical cycling must be started by reduction at the positive electrode (discharge mode). This fact limits the higher charge voltage of the battery. In a full MIB (free of Mg metal), the positive electrode should be able to provide sufficient magnesiation degree of the negative electrode. Ideally, the electrochemical cycling of a full MIB should be started by oxidation at the positive electrode and reduction at the negative electrode (charge mode). Up to now, the spinel-type MgMn_2_O_4_ and related compounds seems to be the most feasible candidate for this type of positive electrode, with a theoretical voltage of ca. 2.8 V. In any case, the positive electrode of the MIB should operate at high-voltage to be competitive, ideally between ca. 2.0 and 4.5 V, and provide high capacity (beyond 120 mAh g^−1^) and high energy density. However, electrode materials with lower voltage and energy density are very often found in the literature. We think that the electrode materials that work below ca. 2.0 V vs. Mg should not be considered as candidate for the positive electrode of any type of practical MIB, and it could be rather a negative electrode for a full MIB.

## 2. High-Voltage Electrode

The positive electrode of a MIB should provide high voltage and allow high mobility of magnesium in its framework. Seeking for that, several types of materials have received the attention of the scientific community. On the other hand, the electrolyte solution must be suitable for both the positive and negative electrode. Unfortunately, the electrolyte solutions which allow the reversible platting of Mg at the negative electrode (near ca. 0 V) are oxidatively decomposed at the high voltage of the positive electrode (over ca. 2.0 V). Thus, the lack of very suitable electrolyte solutions and host materials limits the developing of MIB.

### 2.1. Chalcogenides

If one compares the chalcogenides and the oxides, the larger ionic size of S^2−^ and Se^2−^ compared to O^2−^ could be an advantage for providing wide channels for Mg-diffusion. In addition, the interaction of soft bases, such as S^2−^ and Se^2−^, with the hard acid Mg^2+^ would be weaker compared with the hard base O^2−^. Thus, in principle, the mobility of magnesium in chalcogenides could be better than the mobility in oxides, but this mobility also depends on the crystallographic structure and the diffusion path. For example, the layered-type structure of many transition-metal dichalcogenides (TiS_2_, MoS_2_…) could be useful for the intercalation within its interlayer gap, but the mobility of magnesium in the interlayer usually is rather slow in many cases.

**Figure 5:**
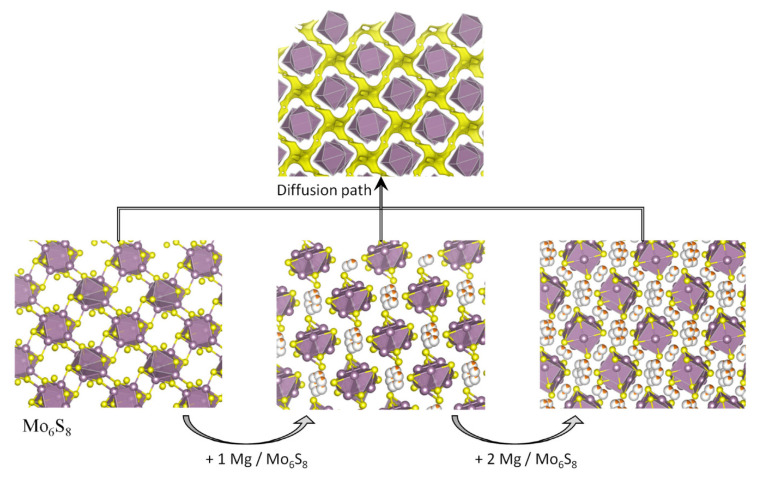
Structural view of the intercalation of magnesium into the Chevrel phase Mo_6_S_8_ (s.g. R $\bar{3}$ ). In the Mo_6_S_8_ unit, six Mo form an octahedron on the faces of a cube, and eight S form the corners of the cube.

Historically, Aurbach’s group reported the Chevrel phase compounds M_x_Mo_6_T_8_ (M = metal and T = S and Se) as the first type of compounds with great potential interest for the positive electrode (cathode) of MIB [21]. The 3D channels in these compounds are useful for the accommodation of many guests and diffusion through the channels. The intercalation of magnesium in this type of materials has been extensively studied, and it seems to be easy and rapid compared to many others electrode materials, and it is also advantageous in terms of cyclability. A structural view of Mg_x_Mo_6_S_8_ is shown in Figure 5. Kganyago et al. theoretically calculated that the maximum voltage for Mg intercalation in Mo_6_S_8_ is 2.23 V [22], but the experimental voltage for Mg intercalation in these materials is substantially lower (ca. 1.1 V vs. Mg). Looking at this voltage, this Chevrel phase could not be considered a good positive electrode for achieving MIB to become competitive against LIB. Nevertheless, according to Ichitsubo et al., the redox potential of some Chevrel compounds containing Cu could be higher (up to 3 V) [23], although the instability of this compound could limit its applicability. The extruding of Cu from the lattice seems to be a common phenomenon in chalcogenides. In addition, perhaps the structure of the Chevrel phase with Mo_6_T_8_ building blocks and channels available for Mg-intercalation could provide the inspiration for finding new materials.

Compared to many other chalcogenides for Mg-intercalation, the Mo_6_S_8_ cathode remains better because its superior electrochemical performance [24]. However, it seems that there is a chance to achieve further progress. Several ways to improve the electrochemistry of the chalcogenides can be the composites, interlayer expansion, nanostructuring, as well as porosity and hollow nanostructure [24]. For example, metalorganic frameworks can be used as templates to synthesize porous nanostructures. For layered-type chalcogenides, Li et al. found that the intercalation of solvated magnesium ([Mg(DME)_x_]^2+^) in MoS_2_-carbon composite is rapid [25].

In the case of the spinel-type TiS_2_, DFT calculations showed that the occupancy of Td and Oh sites by inserted Mg atoms strongly depends on the site of the unit cell. Thus, for diluted Mg content, the Oh site is preferred while for non-diluted concentrations a mixed Oh/Td occupancy is observed [26]. Experimental results confirm the interest of the spinel TiS_2_ with a high capacity of 200 mAh g^−1^ at an average voltage of 1.2 V vs. Mg. On cycling a capacity of 150 mAh g^−1^ is obtained during the firsts cycles with a specific energy is 180 Wh kg^−1^, almost twice that of the Chevrel phase [27].

Carbon coating, polyaniline (PANI) nanoneedles and flower-shape submicrometric MoS_2_ were employed to prepare a MoS_2_@C@PANI composite with a capacity (about 120 mAh g^−1^) superior to pure MoS_2_ [28]. The cycling voltage was between 0.0 and 2.0 V. Thus, the average voltage is low for a cathode.

Multiwalled carbon nanotubes (MWCNTs) and graphene oxide can be added to the chalcogenides to enhance the cycling stability. The nanostructured conductive additives improve the electrical conductivity and buffer the volume change during the electrochemical charge/discharge. According to Zhang et al., the composite CuCo_2_S_4_/CuS@MWCNTs possesses an impressive cycle stability (more than 1000 cycles), although the imposed voltage limits (0.0 and 2.0 V) were low, and the specific capacity is relatively low (around 60–100 mAh g^−1^) [29].

Copper sulfides, such as CuS and Cu_2_S, have high electronic conductivity and can have displacement reactions, but their cycling performance usually is poor because of the structural collapse. These sulfides can adhere on copper foam and employed as binder-free electrode with excellent cycling performance, but relatively low voltage (between 0.0 and 1.8 V vs. Mg) [30]. To the best of our knowledge, the electrochemical behavior of conversion reactions in solid state MIB remains unexplored.

### 2.2. Spinel-Type Oxides

**Figure 6:**
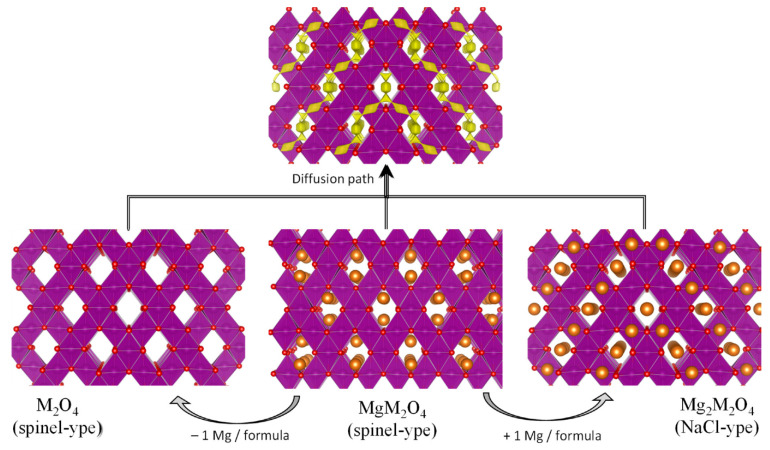
Structural view of the (de)intercalation of magnesium in Mg_x_M_2_O_4_ (s.g. Fd $\bar{3}$ m, except MgMn_2_O_4_ with s.g. I4_1_/amd). The diffusion path is shown at the top.

For a high voltage MIB, it would be desirable that the positive electrode could be directly oxidized in the first charge of the full MIB, suppling Mg to the anode and, for that purpose, the electrode material should contain magnesium in its initial composition. The hypothetical Mg-compounds analogous to the lithium compounds with layered-type structure, such as LiCoO_2_ and LiNiO_2_, are discarded for MIB, because of their intrinsic instability. Thus, the next option is the spinel-type structure with general formula MgA_2_O_4_ (A= Cr, Mn, Fe and Co). It seems that the spinel structure provides a three-dimensional diffusion path which could be suitable for Mg-ion diffusion (Figure 6). A main advantage is that the theoretical and experimental voltage for demagnesiation of these spinels is very high (around 2.8–4.2 V vs. Mg), although it is over the stability window of many electrolyte solutions. In addition, some ions (e.g., Mn^3+^) could catalyze the decomposition of the electrolyte. Thus, the experimental voltage values reported in the literature could be strongly affected by the lack of suitable electrolyte and/or reference electrode.

The compound with structure spinel-type MgMn_2_O_4_ is equivalent to LiMn_2_O_4_. A main difference is that the average oxidation state of manganese is lower in MgMn_2_O_4_ (Mn^3+^) compared to LiMn_2_O_4_ (Mn^3.5+^), and the Jahn–Teller effect of Mn(III) distorts tetragonally the unit cell structure (s.g. I4_1_/amd). The use of MgMn_2_O_4_ in MIB was first published independently by several research groups in 2015 [31,32,33,34]. Since then, this compound and others related compounds have become very promising as electrodes for MIB. The calculated structures of Mg_x_Mn_2_O_4_ are drawn in Figure 6. The theoretically calculated voltage for the demagnesation of MgMn_2_O_4_ and formation of λ-Mn_2_O_4_ is ca. 2.8 V vs. Mg^2+^/Mg, and the theoretical voltage for further Mg-insertion to give rock-salt type Mg_2_Mn_2_O_4_ is ca. 2.0 V [34,35]. It has been found that the Mg-migration in spinel oxides is possible at acceptable rates for small particle size [36], although this migration is sensitive to the structural disorder within the lattice. Gautam et al. found that the magnesium vacancies in MgMn_2_O_4_ open migration pathways and make easier the formation of Mg percolating networks, while the structure inversion is a limiting factor for the electrochemistry [37]. The polarization of the cell, and the hysteresis between the charge and discharge curves are a main concern, although still there is not a very clear separation between the hysteresis due to the behavior of the positive electrode and the hysteresis due to other components of the cell. We have reported that through acid-treatment of MgMn_2_O_4_, cationic vacancies can be created and that these vacancies can decrease the cell polarization and improve the stability of the framework, as it was experimentally observed using symmetric cells and justified by theoretical calculations [35].

As shortcoming for the practical application of MgMn_2_O_4_ could be the thermodynamic instability of the intermediate compositions Mg_1−x_Mn_2_O_4_ (0 < x < 1) which tends to disproportionate, although this process could be slow. A way to overcome this could be the partial replacement of Mn by other elements. Thus, the partial substitution of Mn by iron has been explored to increase the stability of MgMn_2-y_Fe_y_O_4_ during the electrochemical cycling [38,39]. The Jahn–Teller distortion of the structure can be suppressed by incorporating iron to the spinel and the structure becomes cubic (s.g. Fd $\bar{3}$ m). The contraction of the volume of the unit cell upon demagnesiation is smaller when Mn is replaced by Fe. According to the Hull diagram, the intermediate compositions of the Mg_x_Mn_2-y_Fe_y_O_4_ series, such as Mg_0.25_Mn_1.5_Fe_0.5_O_4_, Mg_0.5_MnFeO_4_ and Mg_0.75_Mn_0.5_Fe_1.5_O_4_, are stable, and could allow to achieve further electrochemical cycling. The oxidation of iron from Fe^3+^ to Fe^4+^ would happen at around 3.9–4.0 V, but it probably could not be reversible. Unfortunately, the presence of iron increases the inversion degree of the spinel.

The maximum reversible capacity usually observed for these materials is about 130 mAh g^−1^ [39], although the maximum theoretical capacity is 616 mAh g^−1^ (referred to MnO_2_, and Mg content up to Mg_2_Mn_2_O_4_). Kobayashi et al. raised the experimental capacity of MgMn_2_O_4_ up to 230 mAh g^−1^ by using cubic particles with ca. 5 nm in size and covered with graphene [40]. Tao et al., reported 261.5 mAh g^−1^ at 100 mA g^−1^ of current with long-term cycling stability for hollow spheres of MgMn_2_O_4_ [41].

The redox pairs Co^3+^/Co^4+^ and Ni^3+^/Ni^4+^ could provide very high voltages. Ichitsubo et al. reported an initial open circuit voltage of about 3.5 V for the spinel-type MgCo_2_O_4_ [42]. A rock-salt structure was found for MgNi_2_O_4_. The reasons for the hysteresis observed between the charge and discharge curves of these materials should be further examined.

Another element which can be very useful for partial replacement of Mn in the spinel is Al [43]. In addition, the electrochemical experiments carried out at temperatures above room temperature can decrease the cell polarization and increase the specific capacity up to 270 mAh g^−1^ [38,43], because the Mg-diffusion becomes more rapid at higher temperatures.

**Figure 7:**
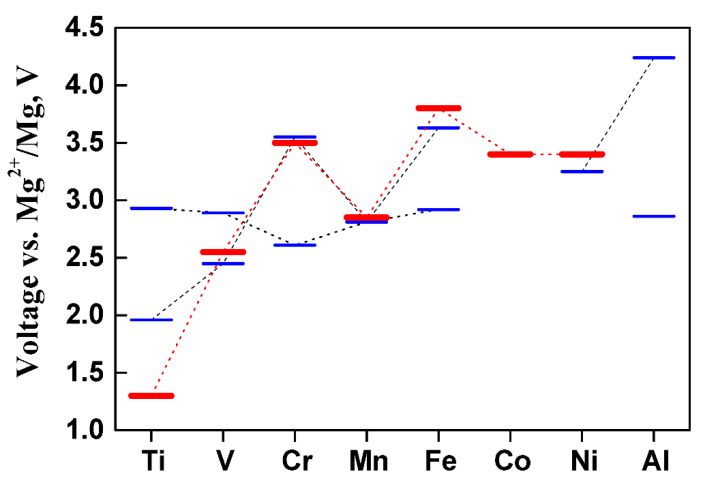
The calculated voltage of some spinel phases as a function of the redox-active transition metal for the reaction M_2_O_4_ + Mg → MgM_2_O_4_. The results in red color were taken from reference [34]. For MgMMnO_4_ with M = Mn and Fe the data were taken from [39]. The results for M = Ti, V, Cr, Ni and Al have been calculated in this work (previously unpublished) using the same method described in [39], with U_eff_ = 3.25 eV (for V), 3.7 eV (for Cr) and 6.45 eV (for Ni) [34].

In general, the choice of the transition elements (M) in MgM_2_O_4_ allows to modify the working voltage of the spinel-type electrode. The lowest value is obtained for M = Ti with ca. 1.3 V, while higher values are obtained for Co, Ni (as already cited) and specially for Fe (Figure 7). In the case of mixed systems, MgMMnO_4_, for M = Ni only one plateau is obtained for Mg extraction, ascribable to the pair Ni^2+^/Ni^4+^. For the others M= Ti, V, Cr, Fe and Al two plateaus are obtained, corresponding to 1 > x > 0.5 and 0.5 > x > 0 in Mg_x_MMnO_4_. The plateau at ca. 2.6–2.9 can be assigned to the redox pair Mn^3+^/Mn^4+^. The other plateau can be ascribable to the redox activity of M: the redox pair M^3+^/M^4+^, being the values for V, Cr and Fe, like those of the non-mixed spinels MgM_2_O_4_ [34]. The exception is for the case M = Ti, where Ti^4+^ is expected in MgTiMnO_4_ and the involved redox pair at ca. 2.0 V can be assigned to Mn^2+^/Mn^3+^. In fact, the calculated voltage for the redox pair Ti^2+^/Ti^3+^ in MgTi_2_O_4_ is ca. 1.3 V [34], while the calculated value for Mn^2+^/Mn^3+^ in the Mg insertion into MgMn_2_O_4_ to give Mg_2_Mn_2_O_4_ is ca. 2.0 V [35].

### 2.3. Molybdenum Oxides

**Figure 8:**
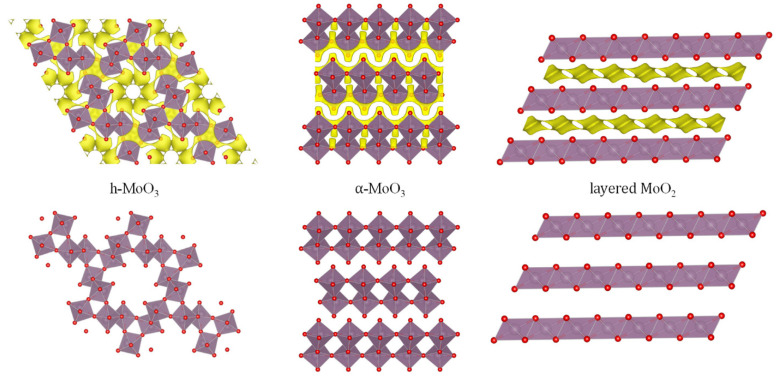
Structure (**bottom**) and diffusion paths (**top**) of different polytypes of molybdenum trioxide: h-MoO_3_ (s.g. P6_3_/m), α-MoO_3_ (s.g. Pnma) and layered MoO_2_ (s.g. C2/m).

Molybdenum trioxide was early proposed as positive electrode for MIB [44]. Interestingly, the theoretical capacity of molybdenum trioxide is 372.3 mAh g^−1^ for 2 F mol^−1^ or 1117 mAh g^−1^ for 6 F mol^−1^. Although there are five polytypes of MoO_3_, the thermodynamically stable phase is α-MoO_3_ (Figure 8-center), which is described as double-layer planar structure, has been much more extensively studied for batteries. According to the web page Materials Project, the calculated intercalation voltage for orthorhombic MgMoO_3_ is 2.2 V, being this theoretical voltage rather low for a MIB [45]. Spahr et al., in 1995 found that the intercalation of Mg into MoO_3_ can be enhanced by using an electrolyte solution containing a small amount of water, and it was explained by the attenuation of the polarization effect of Mg^2+^ by solvating with H_2_O [44]. Gershinky et al. suggested that the intercalation of Mg into α-MoO_3_, at around 1.8 V and with a capacity of ca. 220 mAh g^−1^, drives to the reversible expansion of the interlayer distance [46]. For the better understanding of the rolled played by the Mg-anion pairs, it was calculated that the dissociation Mg-TFSI on the surface of α-MoO_3_ is easier compared to the dissociation of Mg-Cl [47].

It was observed that the ease loss of oxygen from the host lattice of several cathodes is a mechanism of the battery failure. Thus, to increase the stability of α-MoO_3_, using the oxyfluoride MoO_2.8_F_0.2_ was proposed, although the resulting (Mg-intercalation) capacity was only ca. 70 mAh g^−1^ [48]. It was found that the local Mo-anion bonding strength is weakened by replacing O^2−^ with F^−^, facilitating Mg diffusion through the F-substituted lattice. The lowering of activation barrier for Mg-diffusion upon fluorination could be applied to other oxides [49].

The polytype h-MoO_3_ (s.g. P6_3_/m, Figure 8–left), which possesses a structure with wide channels, was studied for Mg-intercalation by Cabello et al. [50]. According to the theoretical calculations using the DFT, the Mg-intercalation voltage is around 2.7–2.9 V, depending on the intercalation site. Thus, the hexagonal polytype could be more promising than the orthorhombic one, in terms of maximum voltage. The insertion of more than one Mg per formula unit can result in the occurrence of Mo metal. Although the theoretical calculations concluded that a large amount of Mg can be intercalated in the tunnels of h-MoO_3_, the experimental results reported by Cabello et al. showed that the liquid electrolyte solution and the counter electrode strongly influence on the apparent electrochemistry and capacity of the MoO_3_ working-electrode. It was proposed that, besides Mg-intercalation, anion (TFSI^−^) adsorption and redox of oxygen in MoO_3_ also can participate in the apparent capacity during the charge-discharge. The layer of TFSI^−^ on MoO_3_ can slow the diffusion of magnesium.

Thus, MoO_3_ is an interesting electrode material for MIB that should be explored with optimized electrolyte solutions and solid electrolytes, and the stability of this material under electrochemical cycling should be further examined.

Besides molybdenum trioxide, layered MoO_2_ (Figure 8-right) also can intercalate magnesium into octahedral sites with theoretical capacity of 279 mAh g^−1^, but the calculated discharge voltage (ca. 1 V) is lower compared to MoO_3_ [51].

### 2.4. Vanadium Compounds

**Figure 9:**
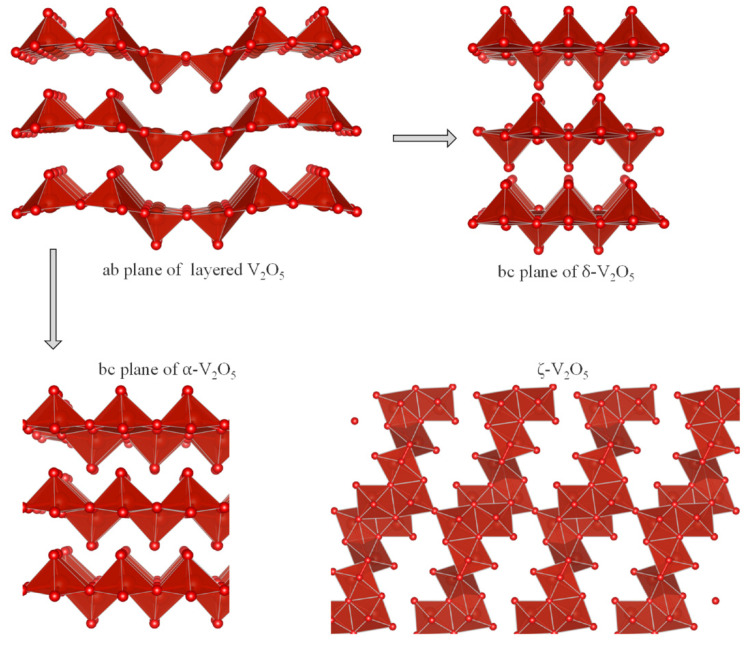
Structures of selected V_2_O_5_ polymorphs: α-V_2_O_5_ (s.g. Pmn2_1_), δ-V_2_O_5_ (s.g. Cmcm) and ξ-V_2_O_5_ (s.g. P2/m).

Besides alloys, Mg-intercalation compounds which operate at low voltages could be used as negative electrode in a full MIB. Compounds based on transition elements with multiple oxidation states, such as vanadium, seem to be promising. On the contrary to that, the toxicity of vanadium could be a drawback. The orthorhombic polymorphs of V_2_O_5_ are among the materials more extensively studied as electrode for MIB [52,53,54,55,56]. The structures of the polymorphs of V_2_O_5_ are based on VO_5_ pyramids (Figure 9). The theoretical energy density of V_2_O_5_ is about 660 Wh kg^−1^, and the electron transfer is related to the V^5+^/V^3+^ redox couple. Avoiding any interference of water and proton impurities, ionic liquid electrolyte has been employed to demonstrate that layered α-V_2_O_5_ (Figure 9-left) is indeed capable of reversibly intercalating magnesium, with a reversible capacity of 280 mAh g^−1^, and without H_2_O or H^+^ cointercalation [57]. Zhou et al. calculated that the hopping barrier for the diffusion of magnesium in α-V_2_O_5_ is 1.26 eV [58], and this value is much higher than lithium one. According to theoretical calculations [53], the δ-V_2_O_5_ phase (Figure 9–top) has better Mg-mobility and higher average voltage (ca. 2.55 V) compared to α-V_2_O_5_, while intercalation of Mg in α-V_2_O_5_ only could happen under very low rates in small particles. Nanosized ζ-V_2_O_5_ (Figure 9 right-bottom) also has been proposed [59]. It is known that V_2_O_5_ in LIB suffers several phase transformations which are harmful for prolonged cycling. The study of the experimental intercalation of Mg in V_2_O_5_ is complicated by the interference of the electrolyte and the other electrodes. For example, Attias et al. reported that DME solvent has a negative impact on the intercalation of Mg in V_2_O_5_ [60]. In addition, the anion TFSI^-^ can react electrochemically with V_2_O_5_ to form a surface film (mainly MgF_2_) which hamper the Mg-insertion [61]. The water makes great impact on the electrochemical behavior. Gautam et al. found that the average voltage of Mg-V_2_O_5_ is ca. 150 mV in a wet electrolyte compared to dry- or superdry-electrolyte [62]. The water-cointercalation and the electrostatic shielding effect of the coordinating H_2_O originate more rapid Mg-insertion, and the presence of water in the electrolyte solution could passivate the Mg anode.

There are several ways to enhance the electrochemical performance of vanadium pentoxide. The amorphization of V_2_O_5_ could be a way to rise its voltage and to enhance Mg-mobility. Arthur et al. prepared amorphous V_2_O_5_ by ball-milling of V_2_O_5_-P_2_O_5_ mixture [63]. The oxygen-vacancies in V_2_O_5-x_, obtained by Ti-doping, could increase the Mg-storage capacity [64]. The intercalation of organic polymers, such as PEDOT and polyaniline (PANI), in the interlayer of V_2_O_5_ has been recently prosed to improve the magnesium storage performance [65,66]. The PANI layer introduced in V_2_O_5_ expands the interlayer spacing, increases the electrical conductivity and gives flexibility to the structure. We think that the organic-inorganic superstructures are very promising for solid state MIB and could make easier the integration of the battery components.

**Figure 10:**
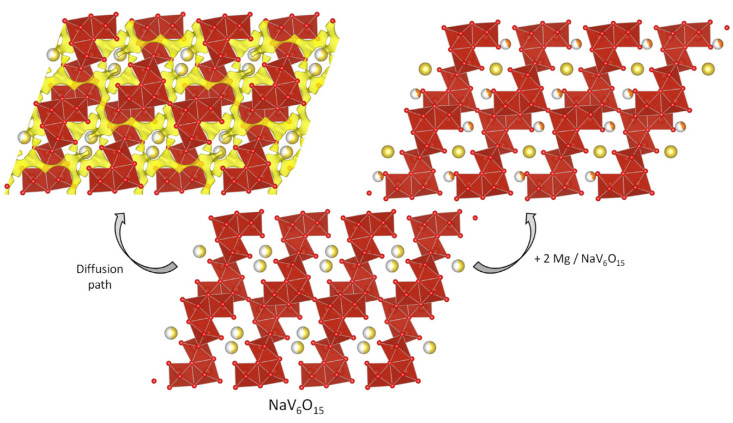
Structural view of the intercalation of magnesium into sodium vanadate NaV_6_O_15_ (s.g. C2/m) and diffusion path.

The tunnels in the structure of β-NaV_6_O_15_ (s.g. C2/m) could be useful for reversible cationic intercalation [67]. Although initially the tunnels are partially occupied by Na, they can accommodate additional Mg atoms, as illustrated in Figure 10. According to the theoretical calculations of Medina et al., Mg-intercalation in β-NaV_6_O_15_ occurs through a plateau at 2.94 V to yield MgNaV_6_O_15_, and another plateau at 1.39 V for Mg_2_NaV_6_O_15_ [68]. Thus, the predicted voltage is interesting for a cathode in MIB. However, the experimental voltage values were lower and strongly depend on the electrolyte solution. The experimental capacity of this material with electrolytes that react with water, such as phenylmagnesium chloride and magnesium borohydride, was negligible. With dry Mg(TFSI)_2_-DME the capacity was 62 mAh g^−1^, and with wet Mg(TFSI)_2_-DME the capacity was around 200–270 mAh g^−1^. The presence of water can increase the capacity of the cathode material, but it could be harmful to the prolonged cycling of the Mg anode electrode. It seems that the sodium atoms remain in the structure of NaV_6_O_15_ and act as “pillars” to stabilize the framework, while Mg is reversibly intercalated. The concept of a full MIB using MgMn_2_O_4_ as positive electrode and β-NaV_6_O_15_ as negative electrode was tested by Cabello et al. [67]. It was found that the difference of voltage between these two electrode materials was not adequate for a commercial battery, at least under the imposed experimental conditions.

By comparison of (NH_4_)_2_V_6_O_16_ and the hydrated form (NH_4_)_2_V_6_O_16_·1.5H_2_O, it is concluded that the lattice water expands the interlayer spacing and promote the migration of Mg in the interlayer [69]. Vanadium can be replaced by Zr, and oxygen vacancies are created, and it may increase the electrochemical performance. Zr-doped ammonium vanadate Zr_0.011_NH_4_V_3.956_O_9.956_ (s.g. C12/m1) can (de)intercalate magnesium in the voltage range between 1.8 and 3.4 V and with a capacity of 328 mAh g^−1^ for 150 cycles [70], and both pure intercalation and pseudocapacitance contribute to this capacity.

Zuo et al. very recently have unveiled that Mg(Mg_0.5_V_1.5_)O_4_ possess a spinel structure (s.g. Fd-3m) [71]. The (de)intercalation of Mg in this spinel was observed in the voltage range between 1.4 and 3.9 V, and the maximum discharge capacity is 250 mAh g^−1^. Even at a high rate (4000 mA g^−1^ of current intensity) the reversible capacity was high (142 mAh g^−1^), and it was sustained over 500 cycles. These authors also tested a full MIB using Mg(Mg_0.5_V_1.5_)O_4_ as positive electrode and Na_2_Ti_3_O_7_ as negative electrode. The electrolyte solution was 0.3 M Mg(TFSI)_2_ in acetonitrile containing 55.9 ppm of water, although the influence of the water in the electrolyte was not discussed.

The use of these vanadium compounds, as well as many others, using solid electrolyte remain unexplored and, since the strong influence of the liquid electrolyte has been reported and the possibility of Mg-intercalation has been observed, it seems to us that the intercalation with solid electrolyte should be experimentally studied. Very probably, the resulting specific capacity would be lower with solid electrolyte, because the contribution of the pseudocapacitance would be reduced.

On the other hand, the stability of the batteries could be improved introducing self-healing electrode materials and/or electrolytes. To gain stable electrochemical performance, the electrode based on V_2_O_5_ was modified with a microcapsule-assistant self-healing [72]. The microcapsules contain a branched polyethylene diamine hydrogel and carbon nanospheres. When the electrode with the active material (V_2_O_5_) particle cracks, the microcapsules would break and release the content of the capsules into the cracks, and the conductive path is repaired. This self-healing allows long-term cycling (1000 cycles) of the MIB. The role played by the water found in the hydrogel was not discussed by the authors.

### 2.5. Phosphates

In 2015, Huan et al. [73] obtained monoclinic V_2_(PO_4_)_3_ by electrochemical delithiation of Li_3_V_2_(PO_4_)_3_ (s.g. P21/c) and then they used the resulting compound as host for magnesium insertion.

**Figure 11:**
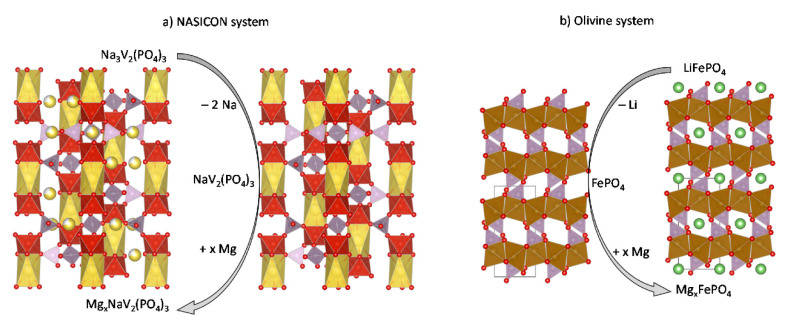
Structural view of the intercalation of magnesium into (**a**) NASICON-type NVP (s.g. R $\bar{3}$ c) and (**b**) olivine-type MTPO_4_ (s.g. Pnma).

Na_3_V_2_(PO_4_) (referred to as NVP) has a rhombohedral NASICON-type structure (s.g. R-3c) and it is a super-ionic conductor of sodium. This type of structure is very stable and can provide topotactic reactions (Figure 11). Cabello et al. found that in Mg cell, sodium is progressively replaced by magnesium during the charge/discharge cycling, probably because the magnesium bond in the host is stronger than the sodium bond [74]. The reaction of the first charge up to 2.2 V vs. Mg is:

Na_3_V_2_(PO_4_)_3_ → Na_1.3_V_2_(PO_4_)_3_ + 1.7Na^+^ + 1.7e^−^ (1)

The overall reaction of the first discharge down to 0.5 V vs. Mg is:

Na_1.3_V_2_(PO_4_)_3_ + 0.4Mg^2+^ + 0.8Na^+^ + 1.6e^−^ = Mg_0.4_Na_2.1_V_2_(PO_4_)_3_ (2)

Thus, both sodium and magnesium are intercalated in NVP. In addition, during the charge/discharge process of these Na-containing compounds, although the initial electrolyte is based on magnesium, a hybrid Na-Mg electrolyte is in situ formed, and the relative concentration of Na/Mg changes with the state of charge. Similarly, Li et al. used a Mg-Na dual salt electrolyte [75].

Zeng et al. reported a reversible capacity of ca. 80 mAh g^−1^ for mesoporous spheres of NVP coated with carbon in MIB after 100 cycles, and the average voltage of the voltage-capacity curve was ca. 2.5 V [76]. Others promising materials of the NASICON-type are Na_3_V_2_(PO_4_)_2_F, Na_1.5_VPO_4_F_0.5_ and Na_2_FeTi(PO_4_)_3_.

Additionally, olivine-type compounds AMPO_4_ are interesting candidates. The lithium iron phosphate LiFePO_4_ is a cathode for commercial LIB. However, there is no structural analogue compound with other ions, such as Na or Mg. Although NaFePO_4_ exists, with maricite structure, it has low conductivity. Thus, a strategy for Na-ion batteries could be to remove Li to yield FePO_4_, and then use it in Na-ion batteries; and the same approach could be used for MIB (Figure 11b):

LiFePO_4_ → Li + FePO_4_ (3)

FePO_4_ + x Mg = Mg_x_FePO_4_ (4)

**Figure 12:**
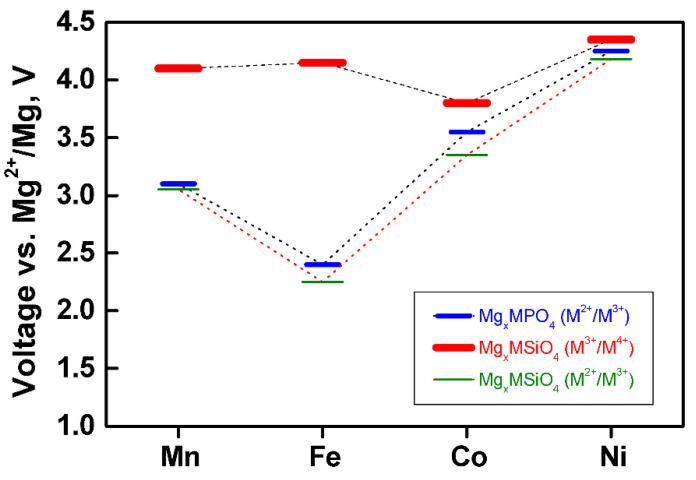
The calculated voltage of some olivine phases as a function of the redox-active element M = Mn, Fe, Co, or Ni [77]. Blue color: M^2+^/M^3+^ in MPO_4_ + 0.5 Mg → Mg_0.5_MPO_4_. Green color: M^2+^/M^3+^ in Mg_0.5_MSiO_4_ + 0.5 Mg → MgMSiO_4_. Red color: M^3+^/M^4+^ in MSiO_4_ + 0.5 Mg → Mg_0.5_MSiO_4_.

Theoretically, the redox pair Fe^2+^/Fe^3+^ imposes a maximum insertion of 0.5 Mg per formula to give Mg_0.5_FePO_4_. The voltage of the reaction depends on the nature of the transition metal (Fe or others), as calculations shown (Figure 12) [77]. For iron phosphate, the hypothetical Mg battery show a suitable voltage within the electrochemical window of the electrolytes, but the voltage is rather low for a cathode [77], while the voltage (near 4 V) is too high for the others.

We can also find some silicates AMSiO_4_ with olivine related structure. The lower oxidation state of Si (as compared with P) allows a higher oxidation state of the transition metal M_T_ up to +4, thus allowing the insertion of 1 Mg per formula:

MSiO_4_ + Mg = MgMSiO_4_ (5)

The capacity increases for silicates, and the voltage of the redox pair M^3+^/M^4+^ is considerably higher than M^2+^/M^3+^ (Figure 12). In fact, it is too high for the electrolyte solutions currently used, but it could be suitable in the future with new electrolytes and with solid state batteries. On the other hand, the redox pair M_T_^2+^/M_T_^3+^ is very similar for phosphates and silicates [77].

## 3. Low-Voltage Electrode

The negative electrode in MIB should be Mg metal or a material which operates near 0 V measured against Mg. Moreover, the electrode materials which operate near at low voltages (below ca. 2.0 V vs. Mg) could not be the positive electrode in a commercial MIB, irrespectively that many papers reported low-voltage electrode materials which were studied as positive electrode in half cells.

### 3.1. Mg Metal

The most obvious material for the negative electrode of MIBs is a Mg foil. The theoretical gravimetric capacity of Mg is 2205 mAh g^−1^, and this large capacity can be a major benefit. In addition, the density of Mg is low (1.74 g cm^−3^). Another main advantage of Mg is its lower propensity towards dendrites formation, compared to lithium, although it was checked that the electrochemical cycling with liquid electrolytes under abusive conditions can drive to Mg-dendrites [78,79]. The main disadvantage of Mg metal is that it reacts with a wide variety of solvents (alkyl carbonates, esters, lactones and nitriles), salts (ClO_4_^−^, PF_6_^−^…) and contaminants (H_2_O, CO_2_…) in the electrolyte solution, and consequently a passivating film is formed on the surface of the Mg electrode which hinders the reversibility of the plating/stripping process [80]. This tendency of Mg results from its low reduction potential. Dou et al. found that the composition of the film on Mg is dominated by organic compounds which do not provide a sufficient electrical insulation and it leads to the decomposition of the electrolyte [81]. The regulation of the interphase between the Mg electrode and the electrolyte solution is critical. Although the traces of water in the electrolyte solution could be advantageous in terms of Mg-intercalation, moisture is harmful for the efficient plating/stripping of Mg, although, in fact, Mg metal is replaced by other materials in many papers. A method to reduce moisture or protic contaminants in the electrolyte solution is to employ BH_4_^−^ [78,79].

Son et al. proposed to use an artificial SEI previously deposited on the surface of Mg [82]. For that purpose, polyacrylonitrile and Mg(CF_3_SO_3_)_2_ were firstly coated on Mg and then heated to 300 °C under inert atmosphere. Another method to tailor the SEI is adding halogen ions into the electrolyte. The Mg surface treated with halogen can help to form a Mg-conducting SEI, but the halogen ions can corrode the current collector, and the polarization of the Mg-electrode usually is significantly increased. Li et al. found that in a Mg-S battery the voltage hysteresis is mainly attributed to the Mg anode, and they confirmed that bromide ion can protect the Mg surface from irreversible reactions with anions (ClO_4_^−^) [83]. Another simple method for creating an artificial SEI on the electrode is by soaking the bare Mg foil in a tetraethylene glycol dimethyl ether solution containing LiTFSI and AlCl_3_, although the SEI can evolve during electrochemical cycling and the overpotential can increase [84]. In the case of the all-solid-state battery, we think that the protective coating on Mg could help to the integration of the anode-solid electrolyte with a good contact at the solid–solid interface, to improve the interfacial stability and to reduce the resistance.

The Mg electrode also can be used in the form of nanometric material with special morphology [85,86]. A new salt that could be highly compatible with the Mg metal anode is magnesium bis(hexamethyldisilazide) [87]. However, the compatibility with high-voltage electrodes should be further examined.

### 3.2. Mg-Alloys

Mg combines with several metallic elements and, thus, the first alternative to the Mg electrode is a Mg-alloy or an intermetallic compound [88]. The Mg-alloy could avoid the detrimental effects of the passivating layer usually formed on pure Mg in contact with many liquid electrolyte solutions [89]. The most suitable metals for that seem to be Bi and Sn, while Si and Ge are discarded, and others should be further examined (Li, Sb, In and Ga). Usually the charge/discharge voltage of the Mg-alloying elements takes place in the region between ca. 0.3 and 0.1 V vs. Mg. A disadvantage is that the theoretical gravimetric capacity is lower compared to pure Mg.

The rhombohedral structure of bismuth makes easy the formation of Mg-Bi alloy at ca. 0.3 V vs. Mg [90,91]. The reaction to produce the Mg-Bi alloy with a maximum Mg-content can be written as:

2 Bi + 3 Mg = Mg_3_Bi_2_ (6)

The Zintl phase Mg_3_Bi_2_ has excellent Mg-conductivity. Interestingly, using bismuth triflate (Bi(OTf)_3_) as additive in the liquid electrolyte can be beneficial for the Mg plating/stripping, and it is attributed to the formation of a Mg-Bi interface with low interfacial resistance [92]. The drawback of Mg-Bi alloy is large volume change, hysteresis in the voltage curve and poor cycling stability. Composites of Bi-nanorods and mesoporous carbon can help to alleviate the volume change and to achieve high capacity (367 mAh g^−1^) and better cycling [93].

The lower atomic weigh of Sn, and the lower voltage of the discharge of Sn vs. Mg (ca. 0.2 V) make the Mg-Sn alloy being more promising than Mg-Bi alloy. Theoretical calculations unveiled that the demagnesiation of Mg_2_Sn occurs at ca. 0.2 V [94]. Unfortunately, the kinetics of the intercalation of Mg in Sn is rather slow. The small particle size of Sn seems to be critical to achieve high capacity [95]. Nacimiento et al. showed as a proof-of-concept a battery using MgMn_2_O_4_ as positive electrode and nanosized-Sn as negative electrode, and the voltage limits of this full MIB were 0.2 and 2.2 V. Thus, the observed average voltage of ca. 1 V is not enough for a competitive MIB, although it opened the possibility of findings other MIB with higher voltages. In addition, the formation of Mg-Sn intermetallics can be studied by using ^119^Sn Mössbauer spectroscopy [95]. To improve the reversibility of the demagnesation/magnesiation, a composite electrode comprising crystalline Mg, amorphous Mg and Mg_2_Sn phases was studied by Ikhe et al., the compatibility of this electrode with several electrolyte solutions was checked [96]. The irreversible dissolution of Mg from crystalline Mg during the first charge creates pores, and these pores decrease the charge transfer resistance. This negative electrode and Mo_6_S_8_ positive electrode were tested in a full MIB, although the average voltage was only ca. 0.9 V. Chen et al. found that the deposition of Mg on the hypoeutectic alloy Mg_14_Sn happens at higher potential and the undesired passivation layer has smaller impedance than the deposition on pure Mg [89]. Using Mg-Sn alloy as negative electrode in full MIB is widespread [70], but the selection of the best electrolyte is still a challenge.

To prepare a very thin foil of Mg is not easy. Maddegalla et al. have proposed to replace the pure Mg foil by an ultrathin (ca. 25 μm of thickness) foil of Mg-alloy (3% Al and 1% Zn), and they concluded that the ultrathin Mg-alloy is much better than the pure Mf foil [97].

### 3.3. Organic Compounds

Compared to inorganic compounds, the investigations on organic compounds for MIB are still relatively rare [98,99]. The main advantages of the organic electrodes are:

(i) Low cost and sustainability.
(ii) The gravimetric capacity, based on light elements, could be very high, compared to compounds containing on heavier elements.
(iii) The mobility of magnesium ion in the organic electrodes could be very high.
(iv) Possibility of fabricating flexible batteries and pouch cells.
(v) The energy storage behavior can be based on dual-ion mechanism [100]. Both the cation (Mg^2+^) and the anion (e.g., ClO_4_^−^) can attach and depart from organic electrodes.

To increase the electronic conductivity of the electrode, a significant quantity of carbon conductive agent must be added to some organic compounds. Interestingly, polymers such as PANI can be both conductive agents and electrochemically active for Mg-storage [66]. In some cases, during the charge/discharge of the electrochemical cells, the organic components of the electrodes can suffer electropolymerization, interfering with the main reaction. In addition, the amorphous character, or poor crystallinity, of some organic electrodes makes difficult to study the mechanism of the reactions.

The main problem of the anthraquinone and derivatives and others organic compounds is the solubility in the electrolyte solution. The anthraquinonyl-based polymers deliver a reversible capacity of around 100- 130 mAh g^−1^ and an experimental discharge voltage of ca. 1.6 V [99].

The enolization redox chemistry in pyrene-4,5,9,10-tetraone (PTO) involves carbonyl reduction (C=O ↔ C-O^−^) at ca. 2.0 V, and this avoids bond cleavage/re-formation and achieves fast cathode redox kinetics compared to inorganic cathodes [101]. To exploit the full power capabilities in PTO, solvents with low viscosity and easy desolvation, such as Mg(CB_11_H_12_)_2_ in tetrahydrofuran, must be employed. Thus, the resulting reversible capacity is 315 mAh g^−1^, and the specific power is 30 kW kg^−1^. A drawback is that a lot of carbon additive (50 wt%) must be used to increase the electronic conductivity, because PTO is not a good conductor.

Polytriphenylamine (PTPAn) is a conductive polymer that can reversibly adsorb ClO_4_^−^ at around 3.0–3.6 V vs. Mg [100]. Perylene diimideethylene diamine (PDI-EDA) reacts with Mg at around 1.3–2.6 V. PTPAn and PDI-EDA can be the cathode and anode, respectively, in a full MIB based on dual-ion mechanism. This organic MIB can operate in the voltage range between 0.01 and 1.6 V with excellent cycling stability (2000 cycles). Rhodizonate salts (e.g., Na_2_C_6_O_6_) have been used as cathode with dual Mg-Li electrolytes and Mg as anode, but the reaction is rather driven by lithium ion [102].

Organic acid anhydrides are another family of electrodes, in which the capacity increases with extending conjugated structure [103]. The red pigment 3,4,9,10-perylenetetracarboxylic dianhydride (PTCDA) exhibits Mg-storage capacity (126 mAh g^−1^) at around 1.5–2.4 V [104]. The reversible electrochemical mechanism involves the transformation between carbonyl groups (C=O) and enolate groups (C-O). Salts (NaCl and KCl) were used to reduce the solubility of PTCDA and to achieve good cycling performance.

As a conclusion, there is much room for developing high-performance organic MIB, and the promising application of that in all-solid state MIB remains few explored.

### 3.4. Others Negative Electrodes

Makino et al. used NASICON-type Mg_0.5_Ti_2_(PO_4_)_3_ as electrode material for magnesium insertion [105], but the Mg-deinsertion was not studied. Although this compound contains magnesium in its composition, the initial oxidation state of titanium (Ti^4+^) in Mg_0.5_Ti_2_(PO_4_)_3_ does not allow starting the electrochemical cycling with an oxidation process, and it very probably it could not be used as positive electrode in a full Mg-ion battery.

The spinel-type Li_4_Ti_5_O_12_ (LTO), known as a “zero-strain” material for LIB, can intercalate Mg with a reversible capacity of 175 mAh g^−1^, corresponding to 1.5 Mg per formula unit of LTO [106]. Although nanosized LTO exhibits very electrochemical cycling, the average voltage of the discharge-charge cycle was only ca. 0.9 V, which is not very useful for commercial MIB.

The capacity values reported for TiO_2_ usually are low, and the charge storage mechanism is rather pseudocapacitive according to recent theoretical calculations [107]. The introduction of Ti-vacancies (22% cationic vacancies) by doping with monovalent anions (F^−^ and OH^−^) allowed to experimentally intercalate magnesium into anatase TiO_2_, and a reversible capacity of about 160 mAh g^−1^ was achieved [108]. Yang et al. have proposed placing protons on the sheets of TiO_2_, and stacking these sheets disorderly [109]. This strategy can assist the dissociation of the Mg-Cl bond, proving fast magnesium intercalation. The ionic conductivity attributed to Mg^2+^ in titanium oxide was as high as 1.8 × 10^−4^ S cm^−1^. The resulting discharge voltage was around 1.0–0.1 V, and the maximum capacity was ca. 250 mAh g^−1^.

Chen et al. studied a full MBI with MgNaTi_3_O_7_ and V_2_O_5_ as electrode materials [110]. For that, firstly MgNaTi_3_O_7_ was prepared starting from layered Na_2_Ti_3_O_7_ (NTO) nanoribbons, and sodium was partially removed from NTO concomitantly to the Mg-intercalation. The authors said that then magnesiated NTO was used as anode, V_2_O_5_ as cathode, and Mg(ClO_4_)_2_-diglyme as electrolyte, and this full MIB was discharged at first and then recharged. Apparently, in the voltage-capacity curves of this full MBI, the plotted voltage was measured vs. Mg and, thus, the veritable voltage of that full MIB is uncertain.

Carbonaceous materials also have been proposed to accommodate magnesium. Although the intercalation of Mg into graphite is thermodynamically unfavorable, Kim et al. proposed that cointercalation of Mg-ether molecules is possible (e.g., 1,2-dimethoxyethane) [111]. This find illustrates the little tendency of some Mg complexes to its desolvation. Very recently, Shimizu have confirmed the co-intercalation of solvated Mg into graphite [112]. Since the removal of Mg from graphite is observed at very high voltage (ca. 3 V vs. Mg) and the cell polarization is very large, it seems impractical to use it in a battery.

Ab-initio molecular dynamics simulation have found that pentagraphene, a new 2D metastable carbon allotrope composed entirely of carbon pentagons [113], could have a capacity of 1653 mAh g^−1^, low Mg-diffusion barrier, and low voltage against Mg [114]. Magnesium ions could be adsorbed in sp^2^-hybridized carbon atoms. These theoretical results should be confronted with experimental results.

## 4. Solid Electrolyte

There are a lot of problems with the use of liquid electrolytes, for example the charge transfer resistance, the corrosive nature of the liquid and the stability window of the electrolyte. All solid-state batteries, where the electrolyte is a solid-state material, can be an alternative to the liquid electrolytes. Solid electrolytes could improve the coulombic efficiency, heat resistance and safety of MIB. The formation of a blocking layer on the surface of Mg would be avoided with solid electrolyte. The main issues are the ionic conductivity of the solid electrolyte at room or moderate temperatures, and the contact between the electrolyte and the electrode. The main ideal characteristics of a solid electrolyte for MIB are [115]:

(i) High ionic conductivity of Mg-ion (>10^−4^ S cm^−1^) at room temperature and/or at moderate temperatures.
(ii) No electronic conductivity.
(iii) Chemical stability within a wide range of temperatures.
(iv) Electrochemical stability in a wide voltage window. The HOMO and LUMO levels must be adjusted at the interface electrolyte/electrode.
(v) Good solid/solid interface properties and good adherence to the electrodes. The poor contacts and the cracking induce current restrictions.
(vi) Low cost.
(vii) Environmentally sustainable.

Finding a material with all these properties is not easy. Although there are relatively few studies on solid electrolytes for MIB, the number of publications about it tended to increase in the last years [115]. The wide experience gained from the ceramic and polymer electrolytes in lithium batteries let us to be optimistic. The main families of compounds used as solid electrolytes are discussed below.

### 4.1. Polymer Electrolytes

Aurbach’s group early published a solid-state battery combining Mg-anode, Mo_6_S_8_-cathode and a gel polymer electrolyte [116]. The gel electrolyte comprised PVdF, Mg(AlCl_2_EtBu)_2_ and tetraglyme. Du et al. reported a gel polymer electrolyte based on polytetrahydrofuran and Mg(BH_4_)_2_ [117].

To improve the ionic conductivity of the polymer gel electrolytes, it is being explored to adding inorganic oxides. Saho et al. reported a nanocomposite polymer electrolyte-based pol(ethylene oxide) (PEO), Mg(BH_4_)_2_ and MgO nanoparticles which provides high coulombic efficiency for Mg plating/stripping, high cycling stability and high efficiency for Mg-intercalation in Mo_6_S_8_ [9]. The same author did not observe reversible Mg plating-stripping in Mg(TFSI)_2_-MgO-PEO polymer electrolyte, and they proposed that the reason for that was the occurrence of the cation complex [Mg(BH_4_)]^+^.

The mechanical flexibility of the polymers offers new possibilities for the solid-state batteries. The polymers also can be used to make coating on the surface of cathode and anode electrodes. A main issue that must be studied in depth is the chemical compatibility between the polymers and the Mg anode [118]. The preparation based on in situ polymerization could improve the interface compatibility [119]. Another shortcoming is the flammability of some organic compounds used as plasticizers in the polymer gel electrolytes.

### 4.2. Phosphates

The NASICON-type phosphates which can provide Mg^2+^-mobility are relatively promising because reasonable conductivities have been found only at temperatures higher than ca. 300 °C. Pioneering works were early published on the diffusion of magnesium in phosphates at high temperatures, because the ionic conductivity at room temperature is low. Thus, Ikeda et al. investigated the ionic conductivity of MgZr_4_(PO_4_)_6_ in the range between 300 and 1000 °C [120]. Imanaka et al. increased the conductivity of MgZr_4_(PO_4_)_6_ up to 6.9 × 10^−3^ S cm^−1^ at 800 °C by doping with Zr, and they found that the reason for the improvement was the formation of Zr_2_O(PO_4_)_2_ secondary phase, and the consequent reduction of the grain boundary resistance [121]. Liang et al. explored the system Mg_0.5_Ce_x_Zr_2−x_(PO_4_)_3_ (x = 0.1, 0.2 and 0.3) and the resulting conductivity was 3 × 10^−6^ S cm^−1^ at 280 °C [122]. It seems that the replacement of Mg^2+^ by Hf^4+^ favors the formation of this structure. Unfortunately, the ionic conductivity of (Mg_x_Hf_1−x_)_4/(4−2x)_Nb(PO_4_)_3_ is practical only at temperatures above ca. 300 °C [123].

### 4.3. Chalcogenides

The higher polarizability of S^2−^ compared to O^2−^ could be favorable to achieve better Mg-mobility. A main concern with the chalcogenide ionic conductors is that often they also have electrical conductivity.

Yamanaka et al. prepared MgS-P_2_S_5_-MgI_2_ glasses and glass-ceramic materials by mechanochemical method and heat treatment, containing P_2_S_6_^4−^ as the main structural units, but the conductivity was only 10^−7^ S cm^−1^ at 200 °C [124]. The glasses can circumvent the grain boundary resistance of the crystalline materials.

Canepa et al. reported the first demonstration of fast Mg-ion at room temperature in a close-packed framework [125]. Via ^25^Mg solid-state NMR, Canepa et al. found that the spinel structure of MgSc_2_Se provides low migration barrier (370 meV). It seems that the good ionic conductivity in this material is also related to the tetrahedral coordination of Mg, the large size of Se^2−^ ion, and the migration of magnesium through a pathway with tetrahedral and octahedral sites. Although this chalcogenide compound with spinel-type structure is promising in terms of its high ionic conductivity (10^−5^–10^−4^ S cm^−1^ at 25 °C), a drawback is related to its high electronic conductivity [12]. This electronic conductivity is originated from point defects in the structure. Perhaps, the doping with other elements and the control of the defect points could help to suppress the electronic conductivity of MgSc_2_Se and finally to find an excellent ionic conductor.

### 4.4. Borohydride

Borohydrides such as Mg(BH_4_)_2_ have low Mg-mobility at room temperature, but it can be increased at moderate temperatures [126]. According to researchers of Toyota Central R&D Laboratory, Mg(BH_4_)(NH_2_) exhibits an ionic conductivity of 10^−6^ S cm^−1^ at 150 °C as due to larger cavities in its structure, and it has and electrochemical window of approximately 3 V [127]. The framework of this compound exhibits two interesting features: a Mg zigzag chain and tunnel structures in the a and b plane. Probably the poor oxidative stability of the hydrides will limit their utility as a solid electrolyte against high-voltage cathodes.

The chelation of magnesium by bidentate ethylenediamine, partially replacing the monodentate BH_4_^−^ ligand, can improve the ionic conductivity [128]. However, the poor electrochemical stability of ethylenediamine limits the voltage window.

### 4.5. Metal-Organic Frameworks

Aubrey et al. proposed a series of metal-organic frameworks (MOF) as a new class of electrolytes, in which the ionic conduction can happen through pores. Room-temperature ionic conductivities up to 2.5 × 10^−4^ S cm^−1^ were found, after impregnating the MOF in solution containing Mg-salt [129]. Park et al. reported a novel mesoporous copper-azolate MOF with a Mg-conductivity of 8.8 × 10^−7^ S cm^−1^ [130].

Interestingly, a film of MOF supported on a porous anodized aluminum oxide can be used as a membrane to separate two different liquid electrolyte solutions and to overcome the incompatibility between the electrolyte solution and the electrode [131]. An electrolyte solution (anolyte) in one side of the cell can be compatible with the anode, and the other electrolyte solution (catholyte) can be compatible with the cathode. Mg-MOF-74 permits selective transport of Mg-ion, blocking solvent molecules and without mixing the two electrolyte solutions for long time.

Yoshida et al. have proposed a new approach to enhance the ionic mobility in MOF [132] The salts of magnesium (e.g., Mg(TFSI)_2_) inside the pores of the MOF change their mobility in the presence of organic vapors. It seems that the MOF is conductor of Mg^2+^ rather than anions (TFSI^−^). Small organic molecules (CH_3_OH and CH_3_CN) are needed for this vapor-induced ionic mobility up to 2.6 × 10^−4^ S cm^−1^ at 25 °C, and this superionic conduction is related to the formation of Mg-complexes in the pores of MOF.

An advantage of the MOF is that the structures and pores can be tailored. For evaluating the MOF as electrolyte, furthers studies on thermal stability and chemical stability against Mg and high-voltage electrodes are required [115]. The economic cost of the preparation of MOF at industrial scale can be another issue.

### 4.6. Other Solid Electrolytes

The antiperovskite-type structure of Mg_3_NAs has been recently suggested as another class of solid electrolyte, and the theoretical calculations found low migration barrier and stability against Mg [133]. The same authors pointed out that the stability of Mg_3_NAs would not be enough against a high-voltage cathode.

An alternative to the conventional electrolytes is a dual layer of liquid electrolyte on the anode and polymer electrolyte on the cathode. With this strategy and properly selecting the materials, the HOMO of the polymer electrolyte could be lower than the Fermi level of cathode, and the LUMO of the liquid electrolyte could be higher than the Fermi level of anode. A prototype quasi-solid-state MIB with this dual layer electrolyte, Mg as negative electrode and perovskite BaTiO_3_ as positive electrode was tested at 55 °C by Sheha et al. [134]. In fact, these authors premagnesiated the perovskite BaTiO_3_, heated the resulting Mg_x_BaTiO_3_ to 150 °C, and then it was used as cathode.

The composite systems Mg(NO_3_)_2_-Al_2_O_3_ and MgSO_4_-Mg(NO_3_)_2_-MgO also have been explored for Mg-ion solid electrolyte, and conductivity values of around 10^−6^–10^−4^ S cm^−1^ at room temperature were reported [135,136]. It was found that amorphous MgO contributes to enhancement of the ionic conductivity.

## 5. Summary and Perspectives

Ideal electrode materials and electrolytes for MIB have not been yet selected, but the recent advancements allow to be optimistic, and it seems that a competitive MIB could be developed. However, the pathway is still full of difficulties. In many cases, the average voltage for Mg-intercalation experimentally determined is lower than the theoretically calculated value. Consequently, the reasons for these discrepancies must be carefully considered in future works. Regarding the theoretical voltage of demagnesiation and the fact that it contains Mg in its initial composition, the most promising cathode material for MIB seems to be the spinel-type MgMn_2_O_4_ and related compounds. However, the experimental study of the electrochemical performance of this material and application are limited by the decomposition of the electrolyte solution. Thus, the influence of the electrolyte composition and the impurities on the electrochemical behavior should be further studied. It is necessary to know the maximum capacity of this material without interferences such as proton-intercalation (due to traces of water in the electrolyte) and polarization due to other electrodes or components of the battery. Another shortcoming is that the Mg-mobility at room temperature is rather low. Common liquid electrolytes are irreversible decomposed at the cathode electrode, and even manganese could catalyze the decomposition. The voltage curves of the electrodes based on vanadium compounds exhibit a lot of hysteresis, and the water molecules influence on the electrochemical behavior. For the phosphates and other compounds that contain Li or Na in its initial composition, it is necessary to further study the intercalation of the uni/divalent ions.

At the anode side, the oxides usually have poor Mg-mobility, and the sulfides can be dissolved in the liquid electrolyte solution during the electrochemical cycling. The reaction between the Mg metal and the liquid electrolyte solution, which limits the efficient and long-term charge/discharge cycling, is one of the main problems of the MIB. Another drawback of the liquid electrolyte is that the great desolvation energy of magnesium in many solvents (e.g., DME) increases the interface impedance. It is necessary to design new strategies to overcome the challenges of ion dissociation, interface resistance and solid-state diffusion.

An approach can be to develop new liquid electrolyte based on chelating, like the multidentate methoxyethyl-amines proposed by Hou et al. [137]. These chelants in the solvation sheath of Mg^2+^ enable easy desolvation, highly reversible Mg anode as well as fast (de)intercalation of magnesium into high-voltage layered oxide cathodes. Thus, the average working of Mg_x_MnO_2_ is ca. 2.5 V, and the capacity is about 270 mAh g^−1^ [137]. The chelant-rich solvation sheaths bypass the energetically unfavorable desolvation process, promote interfacial charge transfer kinetics, reduce the overpotential and eliminate the irreversible reactions for both the anode (Mg) and cathode.

The theoretical volumetric capacity of sulfur is very high (3459 mAh cm^−3^). However, the lack of electrolyte solution compatible with both sulfur and magnesium retards the progress on Mg-S battery. A main drawback is the dissolution of magnesium polysulfides in the electrolyte solution during the electrochemical cycling. Xu et al. very recently have employed Mg(TFSI)_2_ and AlCl_3_ in diglyme as a new electrolyte for Mg-S battery [138]. The sulfur was hosted in Ti_3_C_2_@CoO composite, and it was found that the MXene-type Ti_3_C_2_ is more conductive for magnesium ion, and CoO adsorbs magnesium polysulfides more strongly. The reversible capacity of S-Ti_3_C_2_@CoO is 540 mAh g^−1^, and the average discharge-charge voltage is ca. 1.6 V.

The use of Mg metal and solid electrolyte could be very advantageous and drive to a new generation of batteries. In addition, the possibility of using solid electrolytes could open new pathways to explore properly the intercalation of Mg into many hosts materials, avoiding the shortcomings of the liquid electrolytes. The chemo-mechanical properties of the solid electrolytes, the interface electrode/solid-electrolyte and the ionic transport can be the main issues in all-solid state MIB [139].

The new perspectives and strategies can involve mainly:

(i) Solid electrolytes for avoiding the formation of a Mg-blocking layer on the surface of Mg foil electrode and increasing the safety of the rechargeable batteries.
(ii) Exploring new materials and structures for magnesium solid electrolytes. For example, materials with the perovskite-type structure should be explored.
(iii) Experimental measurements on magnesium mobility in the solid electrolytes.
(iv) Theoretical calculation on accommodation and diffusion of magnesium in solid electrolytes.
(v) Enhancement of the ionic diffusion of the solid electrolyte, for example by reducing the film thickness, creating vacancies and doping.
(vi) Studying the electrochemistry of the most promising electrode materials, previously studied in liquid electrolytes, in new solid electrolytes.
(vii) Developing new methods to synthesize polymer electrolytes.
(viii) Integration of positive and negative electrode, and solid electrolyte, including the compatibility between the electrode and the solid electrolyte and the interface resistance.

It is foreseeable that in the next years the new (liquid and solid) electrolytes and materials very probably will offer opportunities for developing a practical MIB [35,115,137,138,139]. However, it is worth to note that magnesium is a critical raw material, and China has 87% share of global production. The disruptions in the supply chain are leading to record prices, and the current Mg reserves in Europe could be exhausted in November 2021. Since the Mg-processing sectors are now affected, an abundant flow of low-cost magnesium should be guaranteed before the future production of MIB.
